# The fate of some urologic innovations from the last century

**DOI:** 10.1590/S1677-5538.IBJU.2017.05.01

**Published:** 2017

**Affiliations:** Editor Associado, International Braz J Urol Divisão de Urologia do A.C. Camargo Cancer Center Fundação A. Prudente, São Paulo, Brasil

Around the 1980's the external shockwave lithotripsy (ESWL) promoted a revolution in the millenary open surgical approach of urinary stones. After the ESWL, the Endourology procedures and its devices progressed a lot, but several controversies persist in this area, as: What is the best approach during the flexible ureteroscopic lithotripsy, to promote stone fragmentation or dusting? These two visions were put under debate in the Difference of Opinion Section (page 798), respectively by the doctors Meller and Lopes Neto, from Brazil. During this time, ESWL had expanded its applications in in Orthopedics and in Pain Medicine. More recently, new ESWL devices, were developed for new a urologic use: The treatment of erectile dysfunction, but this approach is subject of doubts and some skepticism. To help our readers in understanding this kind of treatment, a Chinese Group performed a review of 15 studies and a metanalisys of 4 controlled randomized trials, focusing in the early treatment results (30 days after intervention) They concluded that low intensity ESWL results in better improvement of erectile function in comparison with the sham treatment groups (page 805).

Returning for urinary lithiasis, to treat small infants usually is a challenge. Colleagues from Istanbul reported their single center experience with the use of miniaturized percutaneous nephrolithotomy (other development of stone management), in 72 children younger than 3years old (page 932).

Other significant modification, verified in the end of the 20th century, was the use of synthetic meshes in urogynecologic surgeries, but after years of use, complications are well known, and resulted in significant amount of legal demands. Gomes et al. evaluated the main complications of the synthetic sub urethral slings utilized in the treatment of female stress urinary incontinence (page 822).

The concept of elective nephron sparing surgeries for the treatment of kidney cancer, was born in the end of the last century, initially for the resection of small tumors (<4.0 cm), and has been progressively consolidated and popularized. Consequently, the indications of partial nephrectomy have been expanded in challenge cases. In this issue of Int Braz J Urol., we have from Pekin, evaluations about the use of partial nephrectomy in pT3a cases; a group from Tel Aviv, reported the outcomes of laparoscopic partial nephrectomy for tumors larger than 7 cm (pages 849-857).

The group of Cleveland Clinic led by Dr. Monga found through the interviews with specific scores, that quality of life of patients with urinary lithiasis is worse than general United States population. With these data, they reinforced the needs of stone prevention measures to improve quality of life of these patients (page 880).

Among the translational papers, there is an interesting study performed in rat models undergone unilateral ureteral obstruction, by the group from Canakkale University, in Turkey. They demonstrated that the Hyperbaric Oxygen Therapy can reduces the functional renal damage changes (measured through biochemical, histological and scintigraphy parameters).

Focal therapy is other new approach of prostate cancer, under intense discussion. Several equipments are in development around the world, project to promote focal gland ablation. In the video section, the Group of L’ Institute Mutualist Montsouris, from Paris, presents a case of focal cryotherapy for patient with anterior prostate cancer (page 995).

Let us following the next urological steps in this century…

**Stênio de Cássio Zequi, MD, PhD** Editor Associado, International Braz J Urol Divisão de Urologia do A.C. Camargo Cancer Center Fundação A. Prudente, São Paulo, Brasil

